# Sex-Dependent Changes in Social Behaviors in Motor Pre-Symptomatic R6/1 Mice

**DOI:** 10.1371/journal.pone.0019965

**Published:** 2011-05-16

**Authors:** Susanna Pietropaolo, Pauline Delage, Sebastien Cayzac, Wim E. Crusio, Yoon H. Cho

**Affiliations:** Institut de Neurosciences Cognitives et Intégratives d'Aquitaine, Université de Bordeaux and CNRS UMR 5287, Talence, France; Alexander Flemming Biomedical Sciences Research Center, Greece

## Abstract

**Background:**

The R6/1 mouse line is one of the most widely employed models of Huntington Disease (HD), a complex syndrome characterized by motor and non-motor deficits. Surprisingly, its behavioral phenotype during the early phases of the pathology when the motor impairments are not manifest yet has been poorly investigated. It is also not clear whether the expression of HD-like symptoms at the pre-motor stage in this mouse model differs between the two sexes.

**Methods:**

Male and female 12 weeks-old R6/1 mice and their wild-type littermates were tested on a battery of tests modeling some of the major neuropsychiatric non-motor symptoms of HD: alterations in social interest, social interaction and communication, as well as disturbances in prepulse inhibition of the acoustic startle response (PPI) and circadian patterns of activity. The lack of motor symptoms was confirmed during the entire experimental period by means of the tail test for clasping.

**Results:**

R6/1 mice displayed marked alterations in all social behaviors which were mainly observed in males. Male R6/1 animals were also the only ones showing reduced body weight. Both male and female transgenic mice displayed mild alterations in the circadian activity patterns, but no deficits in PPI.

**Conclusions:**

These results demonstrate the validity of the R6/1 mouse in mimicking selected neuropsychiatric symptoms of HD, the social deficits being the clearest markers of the pre-motor phase of the pathology. Furthermore, our data suggest that male R6/1 mice are more suitable for future studies on the early stages of HD.

## Introduction

Huntington's disease (HD) is a dominantly inherited neurodegenerative disorder caused by a CAG repeat expansion in the *Huntingtin* gene resulting in a mutated polyglutamine tract in the huntingtin protein [1]. HD is observed in individuals with more than 39 CAG repeats, and it develops through a triad of complex symptoms. Motor impairment and chorea characterize HD pathology, together with weight loss, deficits in cognitive function and psychiatric symptoms [2], [3], [4], [5], [6], [7], [8], [9], [10], [11], [12], [13], [14], [15], [16]. The latter include anxiety and depression [2], [3], [4], [5], [6], [7], [8], altered social behaviors and communication [3], [7], deficits in prepulse inhibition of the acoustic startle response (PPI) [8], and abnormalities in sleep/activity patterns [12], [14], [15], [16]. All symptoms appear usually in midlife and their severity increases progressively, ultimately leading to death [17]. Psychiatric symptoms can be observed with high prevalence at the very early symptomatic phase and represent the very first signs of HD. They are followed by cognitive decline and precede motor impairments by several years [2], [3], [4], [5], [6], [13].

A number of animal models have been developed to study HD, but the R6 mouse lines are the most widely employed [18]. They are based on the integration of the human promoter and exon 1 of the *Huntingtin* gene containing an expanded CAG repeat sequence (approximately 115 repeats for R6/1 and 150 for R6/2 [19]. While R6/2 mice exhibit a juvenile disease onset (around 4–5 weeks of age) [19], [20], R6/1 mice are characterized by an adult onset, developing cognitive deficits around 10–12 weeks of age [21], [22], [23], and motor impairments at 14–20 weeks of age [19], [24], [25]. The adult stage of onset in the R6/1 model (typical of over 95% of human HD cases) and the slower disease progression makes it a valuable tool for understanding the etiopathology of HD [26].

Despite the extensive use of the R6/1 mouse line in the last decade, its pre-motor phenotype has not been investigated in detail. Most of the available studies have focused on cognitive impairments, demonstrating robust deficits in several learning tests in 10–12 weeks-old R6/1 mice [21], [22], [27], [28]. Deficits in both short- and long-term memory were described in pre-motor R6/1 mice in several tasks, including spontaneous [22] and reinforced [27] alternation in a Y-maze, Barnes-maze and object-location learning [22], the water-navigation task [27], and the whisker-dependent sensory-discrimination task [21].

HD-like psychiatric symptoms were also assessed in the pre-motor R6/1 mouse line, but they were limited to anxiety and depression [22], [28], [29], [30], [31]. Pre-motor R6/1 mice were undistinguishable from their wild type littermates in the dark-light [22], [30] and the elevated plus maze [22] tests for anxiety. In contrast, they showed depression-like symptoms in the forced swim and the novelty-suppressed feeding tests [31], although not in the tail suspension test [29], [31]. Altogether, these data suggest the presence of selected emotional alterations in pre-motor R6/1 mice, which appear less robust than the cognitive deficits.

Apart from these cognitive and emotional alterations, to our knowledge no study has investigated the occurrence of early psychiatric symptoms in R6/1 mice. This lacuna is surprising, due to the crucial importance of psychiatric alterations in the initial phases of HD and their deleterious impact on life quality of HD patients and their families. Modeling the complexity of the psychiatric syndrome of pre-clinical HD in an animal model is therefore important both for the designing of novel specific treatments and for increasing our understanding of the neurobiological mechanisms underlying these early markers of HD pathology.

Studies on the R6/1 model so far have employed mice of either sex, and sex differences in the severity of HD-like symptoms in this mouse line have not been systematically investigated. In humans, later onset of motor HD alterations has been described in women [32], while a slightly higher tendency to display neuropsychiatric symptoms was observed in male patients [33], thus suggesting a sex-dependent modulation of the vulnerability to HD. Studying sex differences in HD-like symptoms at the very early stages of the disease in the R6/1 mouse line can therefore be instrumental to enhance the validity of this mouse model.

The present study investigated the expression of HD-like neuropsychiatric symptoms at the pre-motor stage of the pathology in R6/1 mice of both sexes. We hypothesized a sex-dependent vulnerability to the behavioral effects of the HD mutation, as predicted from human data. To test this hypothesis, we assessed alterations in social behaviors (social interest in the three compartment test, social interaction with different types of stimuli, social habituation, and ultrasonic communication), in PPI, and circadian activity patterns.

## Materials and Methods

### Ethics statement

All experimental procedures were in accordance with the European Communities Council Directive of 24 November 1986 (86/609/EEC) and local French legislation.

### Animals

Breeding trios were formed by mating two wild-type C57BL6/J females (IFFA/Credo, Lyon, France) with an R6/1 male (B6.Cg-Tg(HDexon1)61Gpb/J, Stock number: 006471, Jackson Laboratory, Main Harbor, NY, USA; this stock is completely congenic with C57BL/6J with over 12 generations of backcrossing to this background). After 2 weeks the sire was removed and the females were single caged and left undisturbed until weaning of the pups. Mice were weaned at 21 days of age and group-housed with their same-sex littermates (3–5/cage). On the same day, tail samples were collected for DNA extraction and subsequent PCR assessment of the genotypes as previously described [19]. Only litters including male mice of both genotypes were used for experiments. A total of 33 mice were subjected to behavioral testing: 14 males (6 WT and 8 R6/1) and 19 females (9 WT and 10 R6/1).

NMRI mice (7 juvenile males and 10 juvenile females for the first two social tests and 20 adult females for the test of social interaction/habituation) were used as stimulus animals in the social tests. Each stimulus animal was used multiple times in the same experiment (3–4 times in total); its use and order of presentation was always balanced across genotypes. Furthermore, different adult female stimulus mice were employed for the test of social habituation (10 for each sex). The NMRI strain is commonly employed in studies of social behavior, because of its good levels of sociability [34], [35]. Juvenile (3 weeks old) mice of both sexes and adult (12-weeks old) virgin females of the NMRI strain were purchased from Janvier (Le Genest-Saint-Isle, France), housed in same-sex groups, and left undisturbed for a week before being used for testing.

The estrous cycle of the adult NMRI stimulus females was not checked on the day of testing in agreement with previous studies on ultrasonic vocalizations in male and female mice [35], [36], [37], [38], [39], [40], [ see also 41], [42 for reviews], as it has been shown that there is no evidence that the emission of ultrasonic vocalizations is affected by the estrous phase of the stimulus female [43]. In addition, our stimulus mice were most probably not cycling, given the well-known suppression of the estrous cycle in females housed in unisexual groups in the absence of any contact with males [44], [45].

All animals were housed in unisexual groups in polycarbonate standard cages (33×15×14 cm in size; Tecniplast, Limonest, France), provided with sawdust bedding (SAFE, Augy, France), and a stainless steel wire lid. Food chow (SAFE, Augy, France) and water were provided *ad libitum*. The animals were maintained in separate male and female identical colony rooms under temperature (22°C) and humidity-controlled (55%) conditions with a 12∶12 hr light–dark cycle (lights on at 7 a.m.).

### Behavioral procedures

All mice underwent the same battery of behavioral tests that commenced at 12 weeks of age and were conducted as follows. Starting on day 1, the body weight was measured and a three-compartment test for sociability was administered, followed on day 3 by a direct social interaction test with a juvenile of the same sex. On day 5, prepulse inhibition of the acoustic startle reactivity (PPI) was tested followed on day 8 by circadian modulation of locomotor activity. Finally on day 11 a repeated direct social interaction test, but this time with an adult female, was conducted where social habituation and ultrasonic communication were also evaluated. The absence of motor alterations was checked 24 hrs before each test by means of the tail test for clasping [19].

Tests that relied mainly on observations of spontaneous behavior were conducted first in order to minimize possible undesirable transfer effects; tests that involved stressful stimulation (i.e., the acoustic startle test), or required social isolation (i.e., circadian activity and direct social interaction with a female), were conducted last. All behavioral tests were carried out during the light phase of the cycle. For all social tests, conditions of dim illumination (25–30 lux in the center of the testing cage/apparatus) were employed in order to promote exploration and minimize stress and anxiety; furthermore, experimental and stimulus mice were habituated to the experimental room prior to all social tests, being individually housed in standard polycarbonate cages provided with sawdust, food, and water bottles and left undisturbed for about 10–15 min before testing began.

The use of multiple social essays allowed us to detect potential HD-like deficits in different specific aspects of social behavior: the three compartment test provided an evaluation of the preference for a social versus a non-social stimulus with limited possibility of social interaction; to elicit social interest and to avoid adult male-specific aggression, a juvenile of the same sex was employed as the social stimulus. The second social test allowed full social interaction with the same type of social stimulus, i.e., a same-sex juvenile, thus providing not only a quantitative, but also a qualitative evaluation of social investigation. The intruder test with an adult stimulus of the same sex was the only experimental setting that could allow the analysis of ultrasonic vocalizations in adults of both sexes [46]; the latter was accompanied by a full behavioral analysis to evaluate potential concomitant changes in communication and other social behaviors. This intruder test was repeated on 2 trials in order to investigate social habituation.

### Tail test (Clasping phenotype)

The tail test was used to identify animals displaying motor deficits as illustrated by the abnormal clasping of the hind limbs. Mice were suspended by the tail for 10 s; if the mouse acquired a locked body position the result was scored as positive, and was excluded from the data analysis and from subsequent behavioral tests. This procedure allowed us to ensure that only pre-motor HD mice were subjected to behavioral testing.

### Sociability in the three compartment test

#### Apparatus and procedures

The testing apparatus and procedures employed here have been previously described in detail [47]. Briefly, the apparatus consisted of 3 transparent Plexiglas compartments: a central chamber (45×18×25 cm) connected on each side to another compartment (45×20×25 cm) through a small rectangular opening (15×5 cm). Each side compartment contained a round stimulus cage (10 cm in diameter, 7 cm high) made of wire mesh and (hole size: 0.7×0.7 cm) covered by a plastic roof (5 cm high). A metal weight was attached to the roof in order to keep the stimulus cage stable. Each stimulus cage was placed at a distance of 6 cm from the back wall and 4 cm from the sides.

Each experimental subject was introduced into the middle of the central compartment and allowed to explore the apparatus for 2 trials of 5 min each:

Trial 1 (habituation): the stimulus cages were empty; basal levels of exploration were assessed. At the end of this trial the experimental animal was confined in the central compartment by two Plexiglas magnetic doors for 30 sec.
Trial 2 (sociability): a stimulus mouse (4-week old NMRI) of the same sex of the tested subject was placed in one of the stimulus cages, while an object (a plastic grey cylinder, 6 cm in diameter, 2 cm high) was placed in the opposite cage (sides were counterbalanced within experimental groups); both stimuli were presented to the tested animal for the first time, thus controlling for the response to general novelty; preferential exploration of the social *versus* non-social novel stimulus was measured.

#### Variables measured

Tracking images from a camera above the center of the apparatus were analyzed with Ethovision (Version 3.1, Noldus Technology, Wageningen, The Netherlands). Exploration of each stimulus was assessed by measuring the time spent in each compartment containing the stimulus cage. Separate ANOVAs were carried out on each trial with sex and genotype as the between-group factors and compartments as the within-group factors. In trial 2, a sociability score was computed as 100×T_social stimulus_/(T_social stimulus_+T_non-social stimulus_). Finally, the total distance moved in the entire apparatus was measured in each trial.

### Direct social interaction with a same-sex juvenile mouse

#### Apparatus and procedures

Direct social interaction was assessed in a 30×15×14 polycarbonate cage (Tecniplast, Limonest, France) covered by a metal grid and with approximately 3 cm of sawdust on the floor. Procedures were described in detail before [47]. Briefly, each experimental mouse was introduced into the testing cage and left to habituate for 5 min. An unfamiliar stimulus mouse (a 4-week old same-sex NMRI) was then introduced into the testing cage and left there for 3 min.

#### Variables measured

Testing sessions were recorded and videos were analyzed with Observer XT (version 7, Noldus, The Netherlands), taking only the experimental animal into account. One observer who was unaware of the genotype of the animals scored the time spent performing each of the following behavioral categories and elements [48], [49]:

Affiliative behaviors: sniffing the head and the snout of the partner, its anogenital region, or any other part of the body; allogrooming (grooming the partner); traversing the partner's body by crawling over/under from one side to the other.
Nonsocial activities: rearing (standing on the hind limbs sometimes with the forelimbs against the walls of the cage) and digging. Time spent in selfgrooming (the animal licks and mouths its own fur) was analyzed separately, since this is sometimes considered representing a sign of repetitive behavior and emotional distress [48], [50], [51], [52], [53].

### Prepulse inhibition of the acoustic startle reflex

#### Apparatus and procedures

The apparatus (SR-LAB, San Diego Instruments, San Diego, CA, USA) and procedures were previously described in detail [47], [54], [55], [56], [57], [58]. Briefly, animals were acclimatized to the apparatus for 5 min. The first six trials consisted of six pulse-alone trials, two for each pulse intensity (100, 110, or 120 dB_A_), presented in a pseudorandom order. Subsequently, ten blocks of trials were presented. Each block consisted of three pulse-alone trials, one for each pulse intensity, three prepulse-alone trials (+6, +12, or +18 dB units above the background of 65 dB_A_), nine possible combinations of prepulse-plus-pulse trials (3 levels of pulse×3 levels of prepulse), and one no-stimulus trial (i.e., background alone). These 16 trials were presented in a pseudorandom order within each block, with a variable intertrial interval of a mean duration of 15 sec. The session was concluded with a final block of six consecutive pulse-alone trials as in the first block.

#### Variables measured

Reactivity scores obtained on the first and the last blocks of six consecutive pulse-alone trials were separately analyzed to measure startle habituation. The data obtained in the remaining trials were categorized into three main different subsets according to their relevance to distinct behavioral constructs [54], [55], [56], [57], [58]. First, startle reactivity was assessed by the reactivity scores obtained in the intermediate pulse-alone trials. Second, reactivity on prepulse-plus-pulse trials relative to middle pulse-alone trials was used to estimate prepulse inhibition. Third, to measure prepulse-elicited reactivity we included data from prepulse-alone and no-stimulus trials.

To better conform to the assumptions of parametric ANOVA, a natural logarithmic transformation was applied to the startle reactivity scores [47], [54], [55], [56], [57], [58]. PPI was analyzed converting the reactivity data into percent scores (%PPI = 100×(pulse-alone−prepulse-plus-pulse)/pulse-alone) calculated for each subject for each of the nine possible prepulse-plus-pulse combinations and analyzed with pulse and prepulse intensities as the within-group factors.

### Circadian modulation of locomotor activity

#### Apparatus and procedures

The apparatus (Actimeter system, Imetronic, France) was previously described in detail [47]. It consisted of an isolated plastic cupboard containing 8 transparent plastic cages (21×11×17 cm) with a grid floor. A metal food dispenser and a water bottle were inserted in the front wall of each cage, while two horizontal lines of infrared captors (interline distance = 25 mm, distance between two captors = 12.5 cm) were mounted along each of the longer side walls. The cages were illuminated 12 h per day starting from 7 a.m.

#### Procedures

Each mouse was introduced into an activity cage at 5 p.m. and left undisturbed for the subsequent 38 h.

#### Variables measured

Locomotor activity was evaluated based on the number of breaks of the infrared captors. The first two testing hours were analyzed separately, in order to evaluate the locomotor response to a novel environment. The remaining 36 hrs were analyzed in 1 h-blocks with the 12 h-phase as a further within-subject factor, in order to assess the circadian modulation of locomotor activity. Immediately after testing, mice were housed singly in standard cages.

### Direct social interaction with an adult female: habituation and communication

#### Apparatus and procedures

Direct social interaction was assessed in the home cage in which the animals were isolated for about 24 hrs after the previous test of circadian activity. This procedure is commonly employed to evaluate ultrasonic vocalizations in adult mice of both sexes [40], [59], [60]. An unfamiliar stimulus mouse (a 12-week old NMRI female) was then introduced into the testing cage and left there for 3 min. At the end of the first encounter, the intruder was removed and left in a waiting cage for 30 min, before a second 3-min encounter began.

During the test an ultrasonic microphone (Bat detector U30, Ultrasound Advice, UK) set on frequency division 10 was suspended 10 cm above the cage. Vocalizations were recorded using the Spectrogram 15 program (Visualisation Software LLC, sampling rate 48 kHz, format 16 bit) and analyzed with Avisoft SASLab Pro (Version 5. 013, Avisoft, Berlin, Germany) as previously described [47].

#### Variables measured

Testing sessions were recorded and videos were analyzed with Observer XT, as described for the test with a juvenile mouse. Vocalizations were analyzed in terms of frequency and mean duration. Frequencies underwent a square-root transformation to better conform to the assumptions of parametric ANOVA [35], [47], [61].

### Statistical analysis

All data were analyzed by ANOVA with sex and genotype as between-group factors. Within-group factors (e.g., trials, hours) were included as needed. Post-hoc analyses were carried out when appropriate with Fisher's PLSD. Data are presented as mean ± SEM throughout.

All statistical analyses were carried out using SPSS® 13.0 for Windows (Release 13.0.1, SPSS Inc. Chicago IL, USA) and α was set at 0.05.

## Results

### Body weight

R6/1 mice weighed less than their WT littermates [F(1,29) = 20.56, p<0.0001] and this effect was detected in males only [sex×genotype: F(1,29) = 11.53, p<0.01; post-hoc: p<0.05]. The mean (±SEM) values in grams were: males WT: 27.52±0.75, males R6/1: 22.36±0.65, females WT: 20.00±0.61, females R6/1: 19.26±0.58.

### Sociability in the three compartment test

#### Habituation (trial 1)

Animals did not show a preference for any compartment during the first 5 min-trial [all Fs<1, ns; data not shown]. Differences between genotypes were observed for locomotor activity [F(1,29) = 28.32, p<0.0001], R6/1 males being less active than their WT littermates [sex×genotype: F(1,29) = 20.26, p<0.0001; post-hoc: p<0.05].

#### Sociability (trial 2)

**Figure 1:**
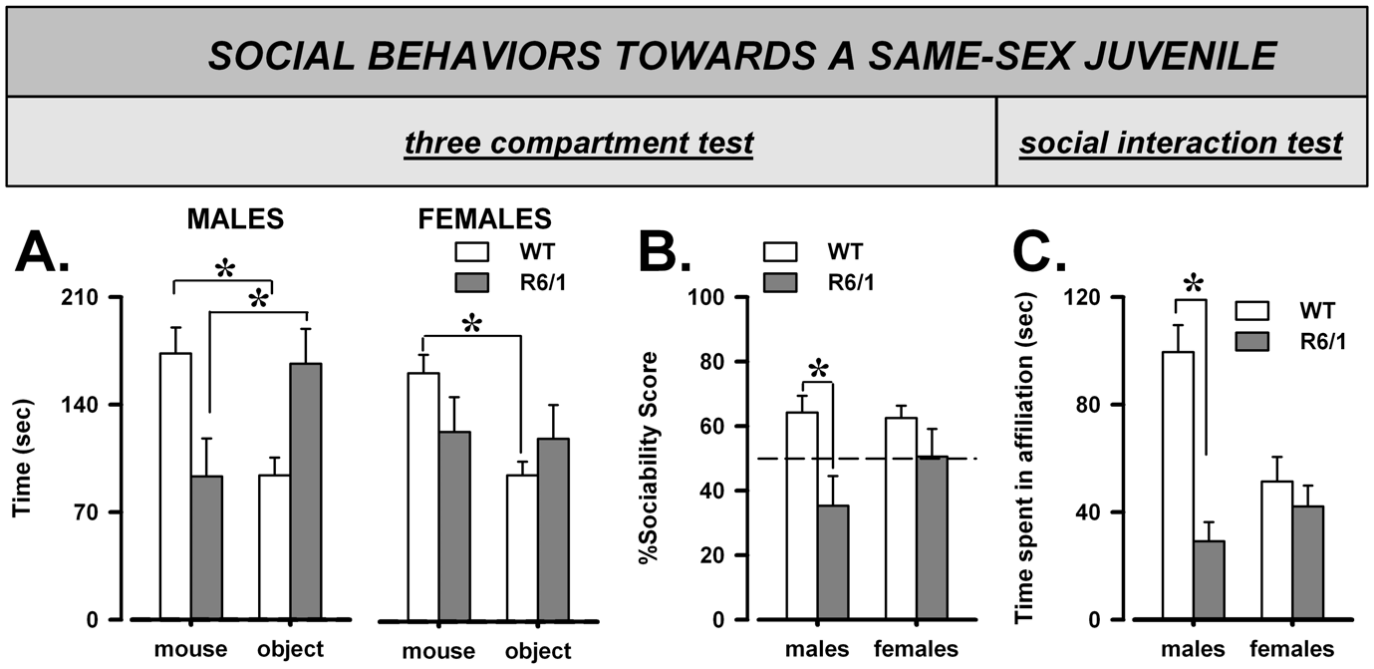
Sociability and affiliation with a same-sex juvenile mouse. Preference for a social (novel juvenile NMRI mouse of the same sex) vs. non-social (novel object) stimulus in the three compartment test. (A) Time spent with stimulus mouse or object, (B) Sociability score. (C) Time spent performing affiliative behaviors during 3 minutes of direct interaction with an unfamiliar juvenile NMRI of the same sex.

Wild-type and R6/1 mice did not differ in their total levels of exploration of the two stimulus compartments [genotype: F(1,29) = 2.48, ns]. However, WT mice preferentially explored the compartment containing the stimulus mouse compared to that with the object, while this preference was absent in R6/1 mice [genotype×compartment: F(1,29) = 7.55, p = 0.01; Fig. 1-B]. The lack of sociability was more pronounced in males, with mutants even showing a preference for the object compared to the social stimulus [post-hoc: p<0.05], while R6/1 females showed no preference for either compartment [post-hoc: p<0.05]. This pattern of results was confirmed by the analysis of the sociability score, supporting the presence of a genotype effect [F(1,29) = 7.30, p = 0.01; Fig. 1-B] which was more pronounced in males [post-hoc: p<0.05].

Locomotor activity differed between genotypes and was higher in R6/1 mice of both sexes [F(1,29) = 7.57, p = 0.01; mean ± SEM (in meters) were: males WT: 1.62±0.13, males R6/1: 1.86±0.11, females WT: 1.49±0.11, females R6/1: 1.88±0.10].

### Direct social interaction with a juvenile male

Data from one female WT mouse were excluded from the behavioral analysis due to technical problems with video recording. R6/1 mice showed lower levels of affiliative behaviors [F(1,28) = 21.61, p<0.0001] and this was observed only in males [sex×genotype: F(1,28) = 12.72, p<0.001; post-hoc: p<0.05; Fig. 1-C]. No difference in any other behavioral measures was observed [data not shown].

### Acoustic startle response

Two males, one WT and one R6/1 exhibited a baseline startle value deviating more than 2 SD from their group mean and were therefore excluded from data analysis [55].

#### Acoustic startle habituation

There was a general reduction in the acoustic startle response from the first to the last block of pulse-alone trials [2-trial block effect: F(1,27) = 7.32, p<0.05], without any differences between sexes or genotypes (data not shown). Startle reactivity increased with pulse intensity [pulse intensity: F(2,112) = 150.1, p<0.0001] irrespective of sex or genotype [sex×pulse intensity and genotype×pulse intensity, ns] and it was overall higher in females [sex: F(1,27) = 5.83, p<0.05].

#### Pulse reactivity on intermediate trials

**Figure 2:**
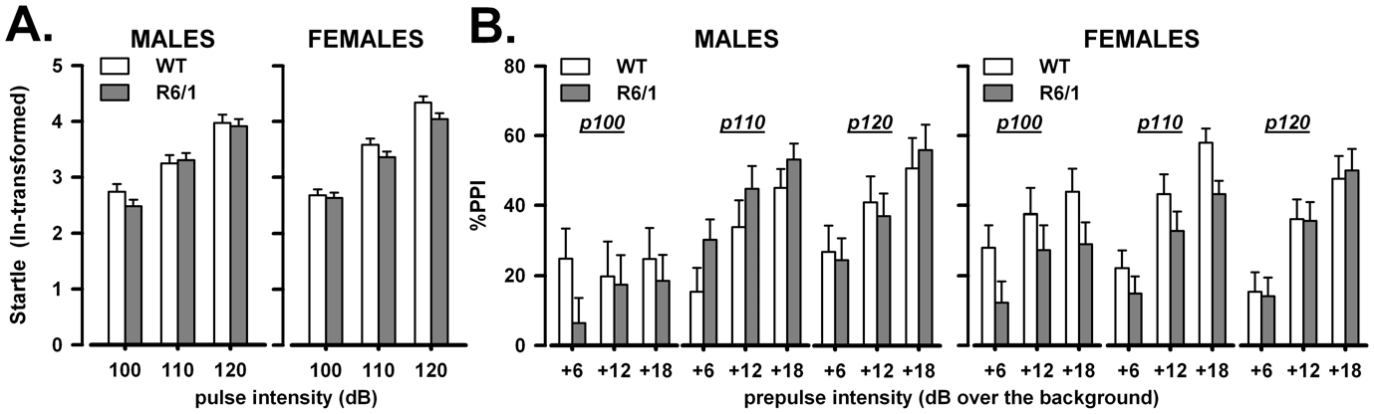
Startle reactivity and PPI (prepulse inhibition of the acoustic startle response). Startle reactivity to the intermediate pulse-alone trials (A). PPI expressed as percentage calculated for each of the 3 prepulse and the 3 pulse intensities (B).

The analysis of the intermediate pulse alone trials did not reveal any differences between the experimental groups, with only a significant effect of pulse intensity [F(2,112) = 200.66, p<0.0001; Fig. 2-A]. As observed for habituation, startle reactivity levels tended to be higher in females, but this difference failed to reach statistical significance here [F(1,27) = 3.33, p = 0.08].

#### PPI

As expected [56], [62], [63], [64], PPI increased with prepulse intensity [F(2,54) = 89.68, p<0.0001; Fig. 2-B] and the magnitude of this effect depended on the pulse level [pulse×prepulse: F(4,108) = 5.49, p<0.001]. While no differences were found between R6/1 and WT mice, PPI was differentially modulated by pulse intensity across sexes [sex×pulse: F(2,54) = 3.23, p<0.05].

#### Prepulse reactivity

The reactivity on prepulse alone trials was also evaluated separately, including trials where only background noise was presented (data not shown). The startle response of all animals increased with the intensity of the prepulse stimulus [F(3,81) = 3.42, p<0.0001] and this effect was similar in both genotypes and sexes.

### Locomotor activity and its circadian modulation

#### Activity and locomotor habituation during the first two testing hours

**Figure 3:**
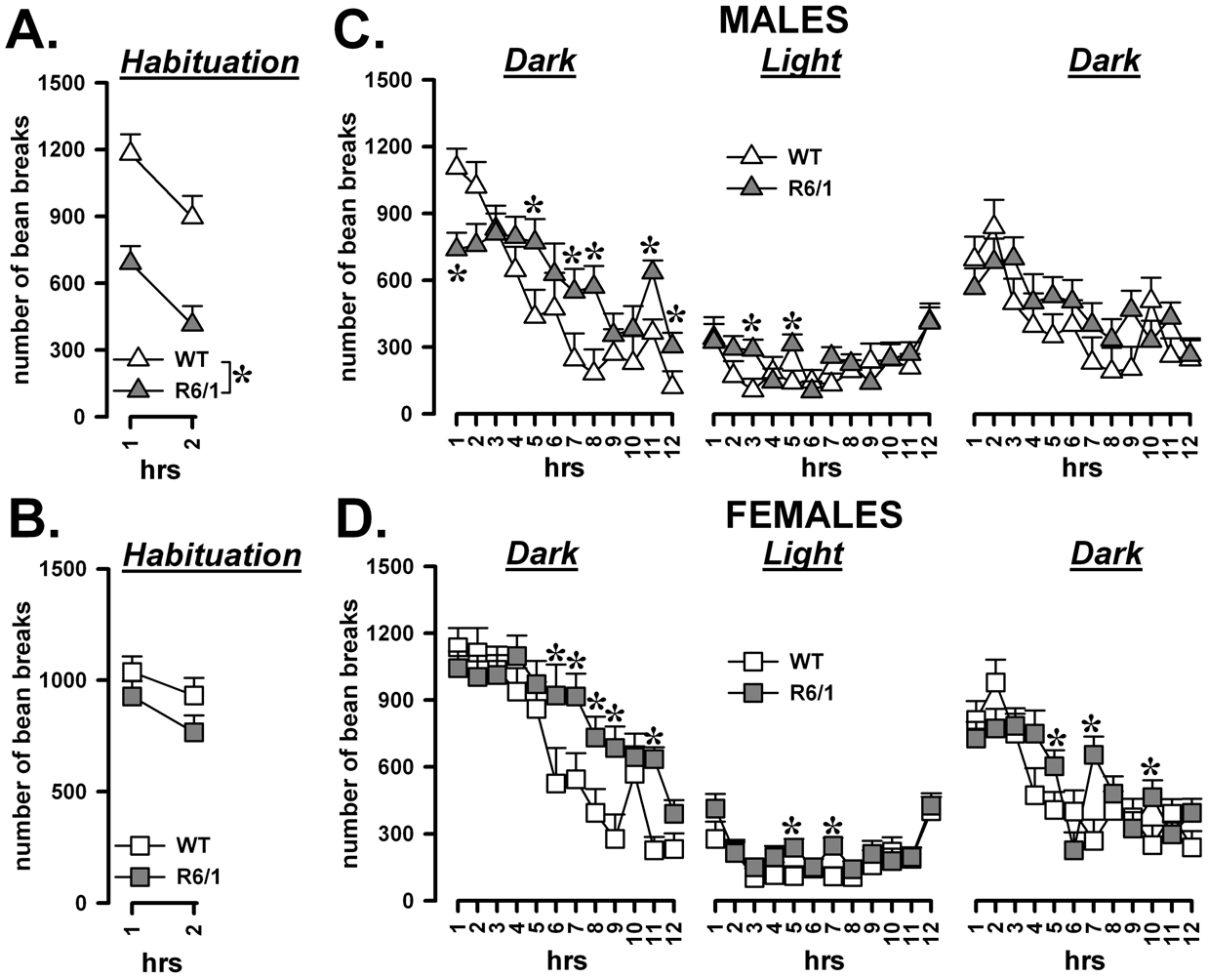
Habituation and circadian patterns of activity. Locomotor response and habituation to a novel environment during the 2 h before the beginning of the first dark phase (A). Circadian modulation of locomotor activity across the subsequent 36 h (B).

The first 2 hours of testing were analyzed separately, in order to evaluate the exploration of the novel environment and locomotor habituation (Fig. 3A–B). Locomotor activity markedly decreased over time [F(1,29) = 29.98, p<0.0001] in mice of both sexes and genotypes. However, R6/1 mice were overall less active during the first two hours than their WT littermates [F(1,29) = 19.22, p<0.001] and this effect was observed in males only [sex×genotype: F(1,29) = 6.06, p<0.05; post-hoc: p<0.05].

#### Changes in locomotor activity during 36 h

The analysis of the subsequent 36 hrs (starting at 7 pm) demonstrated mild but significant differences in the activity patterns of the two genotypes [F(1,29) = 8.68, p<0.01)]. At the beginning of the first period of darkness, R6/1 males were less active compared to WT [sex×genotype×12 hr-period×1 hr-bin: F(22,638) = 1.61, p<0.05; post-hoc: p<0.05; Fig. 3-C], as was already the case during the previous habituation phase. Afterwards, R6/1 mice of both sexes showed subtle disturbances in their activity profile during both the dark and light phases. The activity levels of R6/1 males and females showed a markedly slower decrease over time during the two dark periods – this effect was more pronounced during the first 12 h of darkness – in such a way that the activity levels were significantly reversed by the end of the dark period. The activity of R6/1 mice was not always reduced during the light phase either, where an irregular behavioral profile was observed [genotype×12 hr-period×1 hr-bin: F(22,638) = 2.54, p<0.001; post-hoc: p<0.05; Fig. 3-C, D].

### Direct social interaction with an adult female: habituation and ultrasonic communication

#### Affiliative behaviors

**Figure 4:**
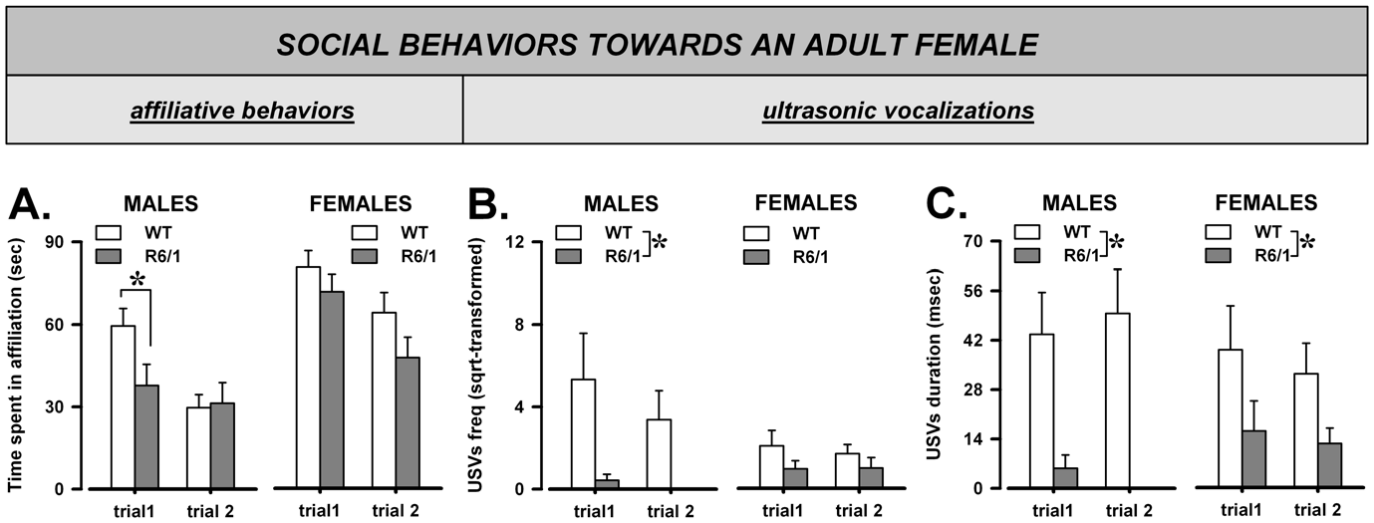
Direct social interaction with an adult female: habituation and communication. Time spent in affiliative behaviors during each of the 3-min trials (A), Frequency (square-root transformed; B), and mean duration (C) of the ultrasonic vocalizations (USVs) emitted during each trial.

As expected, the levels of social interaction decreased across trials, indicating habituation to the social stimulus [F(1,29) = 23.72, p<0.0001; Fig. 4]. This trial effect was observed in all mice, although its magnitude was more difficult to evaluate in R6/1 males, due to their low initial levels of sociability. R6/1 mice indeed tended to display overall lower levels of affiliation [F(1,29) = 3.62, p = 0.07] and this effect was observed mainly in males on trial 1 [sex×genotype×trials: F(1,29) = 3.81, p = 0.06; post-hoc: p<0.05]. No difference in any other behavioral parameters was observed [data not shown].

#### Ultrasonic vocalizations (USVs)

The frequency of USVs decreased across trials [F(1,29) = 7.72, p<0.01; Fig. 4-B], while the mean duration did not significantly change [F<1, ns; Fig. 4-C]. On both trials R6/1 mice displayed a much lower number of USVs than their WT littermates [F(1,29) = 10.38, p<0.01; Fig. 4-B], and this effect was significant only in males, R6/1 mice emitting nearly no vocalizations at all [sex×genotype: F(1,29) = 4.25, p<0.05; post-hoc: p<0.05; Fig. 4-B].

R6/1 animals emitted also USVs of shorter duration than their WT littermates [F(1,29) = 19.21, p<0.001; Fig. 4-C] and this effect was observed this time in both sexes, although it was more pronounced in males [genotype effect in males: F(1,12) = 17.52, p<0.01, in females F(1,17) = 4.46, p<0.05].

## Discussion

The present data demonstrate that the R6/1 mouse line models specific HD-like neuropsychiatric symptoms characterizing the early pre-motor phases of the disease. These alterations were most pronounced in male transgenic mice, especially in social behaviors (Table 1). First, transgenic males displayed social avoidance in the three compartment test where R6/1 females showed only a lack of preference for the social stimulus. Second, only R6/1 males displayed reduced levels of social affiliation, an effect that was observed independently of the type of social stimulus employed. Finally, only male transgenics emitted fewer ultrasonic vocalizations towards an adult female. These results are in accordance with the positive correlation between these two behavioral variables [37], [46]. Interestingly, the mean duration of ultrasonic vocalizations was also reduced this time in R6/1 mice of both sexes, suggesting a qualitative alteration in social communication. Our findings support the notion that analyzing adult ultrasonic vocalizations may be a valuable tool to investigate subtle phenotypic alterations in genetic mouse models for neuropsychiatric disorders [42].

**Table 1:** Summary of the behavioral results.

Type of HD-like symptoms	Behavioral domain	Behavioral Test	Effects of the R6/1 mutation	Presence in R6/1 MALES	Presence in R6/1 FEMALES
social	social interest	three compartment	lack of social interest	+	+
social	social interaction	interaction with a same sex juvenile	reduced affiliation	+	−
social	social interaction	interaction with an adult female	reduced affiliation	+	−
social	communication	interaction with an adult female	deficits in ultrasonic vocalizations	+	+/−
non-social	circadian activity	38 hrs-activity monitoring	mildly altered activity patterns	+/−	+/−
non-social	acoustic startle and its plasticity	startle test	normal startle, habituation and PPI	−	−

Social and non social symptoms were detected in R6/1 mice, Social HD-like deficits were mostly observed in R6/1 males. + = present, − = absent, +/− = partially present, i.e., detected only on duration (for ultrasonic vocalizations) or on some time-bins (for circadian activity).

It is important to note that these alterations in social behaviors were not confounded by reduced levels of general exploration or activity: during trial 2 of the three compartment test R6/1 mice displayed higher levels of locomotion and a similar overall exploration of the social and non-social stimuli compared to their wild type littermates. Furthermore, during all other social tests, transgenic animals did not differ from wild types in the expression of non-social activities.

Although caution is always necessary when translating results from mice to humans [26] and no exact match can exist between these two species, it is clear that the social abnormalities shown by R6/1 mice (especially males) can parallel the social apathy and withdrawal, as well as the deficits in verbal fluency observed in HD patients in the very early phase [2], [3], [4], [5], [6], [13]. Future studies on other specific aspects of social behavior, e.g., social reward, will be important to further characterize the social apathy-like phenotype of the R6/1 model. Further investigation is also needed to clarify whether these HD-like social deficits are due to specific brain alterations that differ from those underlying other psychiatric symptoms, an issue that has never been assessed either in humans or in HD mice. The social deficits observed here could be related to the early alterations in striatal functionality that have been demonstrated already at the motor pre-symptomatic stage in this mouse model [65], [66], as in mammals this brain region is implicated in the modulation of social behavior [67]. Furthermore, the striatum is functionally connected to other brain regions also involved in the control of social behaviors, such as the limbic system [68]. Dysfunctions in other brain regions could also contribute to the observed social deficits: for example, a down-regulation of the endocannabinoid system –known to be involved in the control of social behaviors [69]- has been detected in several brain areas of pre-symptomatic R6/1 mice, including the limbic system [29], [30]. Whether all these brain alterations are specifically responsible for the social deficits of R6/1 mice is a hypothesis that needs to be specifically tested. Pharmacological studies could also contribute to understand the mechanisms underlying the social deficits displayed by R6/1 mice, for example by comparing the effects of treatments already used in HD patients, e.g., targeting the dopaminergic, serotonergic, or glutamate systems [70], [71], or reducing oxidative stress [72], on social and non-social HD-like psychiatric symptoms in these mice. Longitudinal studies including more advanced ages are also warranted to evaluate whether the appearance of social deficits in the R6/1 mouse evolves together with other non-social psychiatric deficits (like in humans) and if it is stable or exacerbates across time.

It is likely that the sex differences observed here in the expression of social HD-like symptoms reflect differences in the severity of early HD-like dysfunction. This hypothesis is supported by previous studies in other genetic mouse models of HD, demonstrating a higher striatal susceptibility to oxidative stress in males [73]. Estrogens have been also proposed to contribute to a reduction in the vulnerability to HD of female mice, either directly, by acting on striatal neurotransmission [74], or indirectly, through their neuroprotective effects, e.g., by upregulating neurotrophic factors [75], [76].

Differences in the severity of early HD-like pathology could also explain the sex difference in body weight loss, observed only in R6/1 males. Weight loss is indeed a characteristic of HD patients, despite increased caloric intake [77]. Although the exact molecular bases of this symptom are still unknown, recent studies have demonstrated the presence of alterations in adipose tissue function in HD patients [78], while reduced circulating levels of insulin-like growth factor (IGF-1) have been found in patients and in R6/2 mice [79]. It is possible that some of these pathological mechanisms develop earlier in male than in female R6/1 animals, a hypothesis that should be specifically tested in future studies.

To our knowledge, this is the first study demonstrating sex-differences in pre-symptomatic changes in the R6/1 model for HD, although comparing our results with previous ones is difficult, since most studies on the R6/1 model have employed either only male or female animals or, when mice of both sexes were used, did not examine or describe the interactions between genotype and sex [80] [81]. However, a previous longitudinal study in the 140-CAG KI model of HD demonstrated that the onset of motor behavioral alterations occurred earlier in male than in female mice [74], in agreement with some human data [32]. Our results therefore are in agreement with the findings that males are more vulnerable to the behavioral effects of the R6/1 mutation and show that this sex difference also affects the expression of non-motor HD-like symptoms.

In contrast to the sex-dependent and marked social deficits, the non-social behavioral alterations in R6/1 animals observed here were similar in both sexes and of mild severity only. The changed circadian activity patterns were subtle, but resembled those observed in HD patients, e.g., prolonged latencies to sleep interrupted by frequent awakenings [12], [14], [15], [16]. Furthermore, no deficits in PPI, startle reactivity, or its habituation were observed in R6/1 mice. To our knowledge, PPI deficits have been evaluated in HD patients only at the late stages of the disease, when startle reactivity and habituation were still normal [8]. Combined with our results, this suggests a specific reduction in sensori-motor gating, but only in the later stages of the disorder. Further studies are needed, however, to assess whether the PPI deficits are limited to the advanced phase of the pathology or whether the R6/1 mouse line does not model this HD-like symptom.

In conclusion, we have shown that the R6/1 mouse models selected neuropsychiatric symptoms of HD, namely alterations of social behaviors, which appear to be valuable markers of the pre-motor phase of HD pathology. In addition, as in humans, male R6/1 mice appear to be more vulnerable to the early effects of the HD mutation than females.
